# IFEM model curriculum: emergency medicine learning outcomes for undergraduate medical education

**DOI:** 10.1186/s12245-024-00671-9

**Published:** 2024-08-05

**Authors:** Arif Alper Cevik, Elif Dilek Cakal, James Kwan, Simon Chu, Sithembile Mtombeni, Venkataraman Anantharaman, Nicholas Jouriles, David Teng Kuan Peng, Andrew Singer, Peter Cameron, James Ducharme, Abraham Wai, David Edwin Manthey, Cherri Hobgood, Terrence Mulligan, Edgardo Menendez, Juliusz Jakubaszko, International Federation for Emergency Medicine Undergraduate Emergency Medicine Learning Outcomes Update Collaborators, Abdullah Abdulkhaliq Qazzaz, Aisha Hamed Al Khamisi, Amal Al Mandhari, Amber Hathcock, Aus N. Jamil, Borwon Wittayachamanakul, Bret Nicks, Carlos E. Vallejo-Bocanumen, Cem Oktay, Chih-Hsien Chi, Conor Deasy, Craig Beringer, Doris Lorette Uwamahoro, Dorota Rutkowska, Erin L. Simon, Faith Joan Gaerlan, Frida Meyer, Immad S. Qureshi, Janet Lin, Jesús Daniel López Tapia, Justin Kaplan, Keamogetswe Molokoane, Kuldeep Kaur, Lars Petter Bjoernsen, Lisa Kurland, Matthew Chu, Miklos Szedlak, Mohamed Alwi Abdul Rahman, Mohan Kamalanathan, Ndebwanimana Vincent, Oscar Navea, Pariwat Phungoen, Pauline F. Convocar, Peter Vass, Philipp Martin, Rahim Valani, Richard Henry S. Santos, Ruth Hew Li-Shan, Sabrina Berdouk, Saleem A. Varachhia, Sam Thenabadu, Sameer Thapa, Sean Kivlehan, Sofia Basauri, Syed Ghazanfar Saleem, Valerie Krym, Victor Lee, Wee Choon Peng Jeremy, Zsolt Kozma

**Affiliations:** 1 Emergency Medicine Section, Department of Internal Medicine, College of Medicine and Health Sciences, United Arab Emirates University, Al Ain, United Arab Emirates; 2 Department of Emergency Medicine, Tawam Hospital, Al Ain, UAE; 3 Emergency Department, Leicester Royal Infirmary, University Hospitals of Leicester NHS Trust, Leicester, UK; 4 Department of Emergency Medicine, Tan Tock Seng Hospital, Singapore, Singapore; 5 Yong Loo Lin School of Medicine, National University of Singapore, Singapore, Singapore; 6 University of Adelaide, Lyell McEwin Hospital, Elizabeth Vale, SA Australia; 7 Department of Emergency Medicine, University of Namibia, Northern Campus, Oshakati, Namibia; 8 Department of Emergency Medicine, Singapore General Hospital, Singapore, Singapore; 9 Department of Emergency Medicine, Northeast Ohio Medical University, Rootstown, Ohio USA; 10 Australian Government Department of Health and Aged Care, Canberra, ACT Australia; 11 Australian National University Medical School, Acton, ACT Australia; 12 Department of Epidemiology and Preventive Medicine, School of Public Health and Preventive Medicine, Monash University, Clayton, Australia; 13 The Alfred Hospital, Emergency and Trauma Centre, Melbourne, Australia; 14 McMaster University, Hamilton, Ontario Canada; 15 Department of Emergency Medicine, School of Clinical Medicine, The University of Hong Kong, Hong Kong, Hong Kong; 16 Department of Emergency Medicine, Wake Forest School of Medicine, Winston Salem, NC USA; 17 Department of Emergency Medicine, University of North Carolina School of Medicine, Chapel Hill, NC USA; 18 Department of Emergency Medicine, University of Maryland School of Medicine, Baltimore, MD USA; 19 Department of Emergency Medicine, Churruca Hospital UBA, Buenos Aires, Argentina; 20 Department of Emergency Medicine, Wroclaw University of Medicine, Wroclaw, Poland; 21 International Federation for Emergency Medicine, Melbourne, Australia

**Keywords:** Emergency medicine, Medical school, Medical students, Undergraduate training

## Abstract

**Background:**

The International Federation for Emergency Medicine (IFEM) published its model curriculum for medical student education in emergency medicine in 2009. Because of the evolving principles of emergency medicine and medical education, driven by societal, professional, and educational developments, there was a need for an update on IFEM recommendations. The main objective of the update process was creating Intended Learning Outcomes (ILOs) and providing tier-based recommendations.

**Method:**

A consensus methodology combining nominal group and modified Delphi methods was used. The nominal group had 15 members representing eight countries in six regions. The process began with a review of the 2009 curriculum by IFEM Core Curriculum and Education Committee (CCEC) members, followed by a three-phase update process involving survey creation [The final survey document included 55 items in 4 sections, namely, participant & context information (16 items), intended learning outcomes (6 items), principles unique to emergency medicine (20 items), and content unique to emergency medicine (13 items)], participant selection from IFEM member countries and survey implementation, and data analysis to create the recommendations.

**Results:**

Out of 112 invitees (CCEC members and IFEM member country nominees), 57 (50.9%) participants from 27 countries participated. Eighteen (31.6%) participants were from LMICs, while 39 (68.4%) were from HICs. Forty-four (77.2%) participants have been involved with medical students’ emergency medicine training for more than five years in their careers, and 56 (98.2%) have been involved with medical students’ training in the last five years. Thirty-five (61.4%) participants have completed a form of training in medical education. The exercise resulted in the formulation of tiered ILO recommendations. Tier 1 ILOs are recommended for all medical schools, Tier 2 ILOs are recommended for medical schools based on perceived local healthcare system needs and/or adequate resources, and Tier 3 ILOs should be considered for medical schools based on perceived local healthcare system needs and/or adequate resources.

**Conclusion:**

The updated IFEM ILO recommendations are designed to be applicable across diverse educational and healthcare settings. These recommendations aim to provide a clear framework for medical schools to prepare graduates with essential emergency care capabilities immediately after completing medical school. The successful distribution and implementation of these recommendations hinge on support from faculty and administrators, ensuring that future healthcare professionals are well-prepared for emergency medical care.

## Background

All medical graduates should be capable of providing basic emergency care after medical school regardless of where they work or the type of medical practice they provide [1]. However, the level of basic emergency care expected from medical graduates varies among countries and healthcare systems. Accordingly, there is little agreement on what, when and how to teach basic emergency care during medical student training [2–7]. International consensus guidelines can help to maintain and advance acute care standards, particularly in the early stages of emergency care development. However, documents to guide emergency medicine education for medical students are scarce [2, 3, 7, 8].

To address this gap, the International Federation for Emergency Medicine (IFEM) published the first international model curriculum for medical student education in emergency medicine [3]. In this endeavour, IFEM provided recommendations regarding learning objectives, unique content areas for emergency medicine, and general undergraduate emergency medicine curriculum content to support high-quality acute care worldwide by setting the standards of basic emergency medicine education [3]. This model curriculum served as an inspiration for many national or institutional emergency medicine curricula for medical students [9–12].

Despite its global impact over a decade, we anticipate that some circumstances might affect the adaptation of the original model curriculum. The principles of emergency medicine and medical education continue to evolve based on societal, professional, and educational needs and advancements. The function of acute care education in medical school differs among systems due to the roles given to medical graduates in the provision of healthcare [7, 13–15]. Diversity in the duration and educational systems of medical schools, the structure and implementation of emergency medicine education, and healthcare systems in different settings exacerbate the necessity of revisiting IFEM’s first model curriculum and providing setting- and resource-neutral Intended Learning Outcomes (ILOs).

IFEM Core Curriculum and Education Committee (CCEC) Undergraduate Emergency Medicine Curriculum Update Taskforce believes that identifying the minimum emergency care-related capabilities of a medical graduate immediately after medical school (i.e., Intended Learning Outcomes - ILOs) can provide all medical schools in diverse systems and settings with a focused and transparent basis to build subsequent curriculum components such as teaching and learning activities, assessment methods, and curriculum evaluation [16, 17]. Accordingly, this report aims to identify emergency medicine-related learning outcomes for medical student education based on available resources using consensus methodology.

## Methods

### Study design

This exercise employed a consensus methodology with a combination of two methods of nominal group and modified Delphi.

**Fig. 1:**
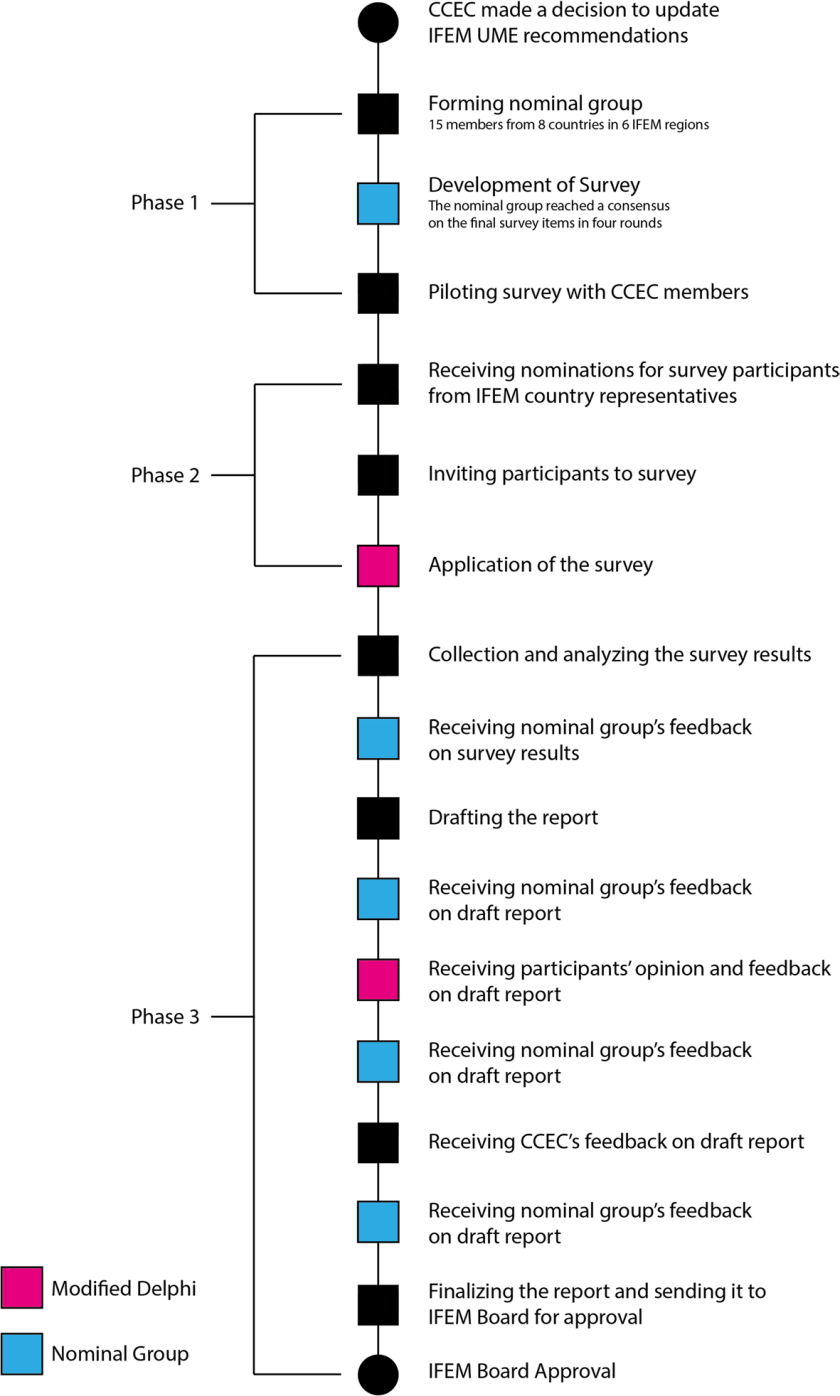
Process Flowchart

In September 2018, two IFEM CCEC members reviewed the 2009 IFEM Undergraduate Medical Education Curriculum and recommended an update plan to encompass advancements in understanding of emergency medicine and medical education. The update process included three phases (Fig. 1), as explained below:

#### Phase 1: The creation of a nominal group and survey development (Nominal Group Technique)

The goal of the first phase was to develop a survey based on the consensus of a nominal group of international representatives. Nine CCEC members from four countries in three IFEM regions (Asia, Australasia, and North America) stepped forward to form the initial nominal group. To increase the diversity of educational perspectives and inclusivity of the final document, six additional emergency medicine experts were invited to the nominal group in a way to represent all six IFEM regions (Africa, Asia, Australasia, Central/South America, Europe, and North America). The final nominal group had 15 members representing eight countries in six regions.

The nominal group reached a consensus on the final survey items in four rounds. For the first round, two investigators reviewed the literature and created a draft survey containing six sections and 487 preliminary items [3–6, 10, 18–22]. The draft survey was introduced to the nominal group in a synchronous online meeting. After careful consideration, the nominal group deemed three sections (chief-complaint-based content, specific diseases and topics, and skills content) categorically less essential and relevant to the decision-making process of the ILOs as including these items would potentially lengthen the survey without clear benefit, decreasing the response rate and reducing the generalizability of recommendations in various settings. As such, 448 items across these three sections were removed from the survey. Then, the remaining items were sent to the nominal group via an online form to collect quantitative and qualitative feedback. Regarding quantitative feedback, the nominal group voted on items to have them excluded. Any item that reached an 80% exclusion vote was removed from the survey. Additionally, open-ended comments were obtained regarding face and content validity, comprehensibility, and comprehensiveness. Two investigators revised the draft survey structure and items based on votes and comments collected. In the remaining second to fourth rounds, the nominal group reviewed the revised versions from the previous rounds until the consensus was reached at the end of the fourth round.

In July 2020, the consensus version of the survey was sent to 34 IFEM CCEC members from 13 countries in 5 regions to pilot test survey for clarity and comprehensiveness. A reminder was sent to non-responders after two weeks. Additional minor refinements were made based on feedback provided by CCEC members to reach the final version of the survey.

### Survey content

The final survey document included 55 items in 4 sections, namely, participant & context information (16 items), intended learning outcomes (6 items), principles unique to emergency medicine (20 items), and content unique to emergency medicine (13 items). Each section was preceded by a short explanation referring to the section’s aim, scope and instructions. Additionally, an area for open-ended comments was provided to participants at the end of the survey. For each item in sections titled intended learning outcomes, principles unique to emergency medicine and content unique to emergency medicine, participants were requested to select one between three options of “must have,” “good to have,” and “not applicable.” “Must have” was reserved for the items that are considered “indispensable to undergraduate emergency medicine training” by the participant. “Good to have” was reserved for items that are considered “should be a part of undergraduate emergency medicine curriculum but is not a priority.” Not applicable was reserved for items that are considered “not relevant to undergraduate emergency medicine curriculum” by the participants. The final survey took approximately 10 minutes to complete and is available for review at the link.

The survey document was preceded with a cover page, including the explanations about the scope of the survey and instructions to participants. The participants were requested to imagine “what a medical graduate should be capable of immediately after medical school.” "Medical graduate" was defined as recently graduated medical students on their first day of work as a doctor, which could refer to various job titles in different contexts, including intern/house officer, resident/registrar or general practitioner. Participants were requested to focus on emergency medicine-related capabilities that medical graduates must have acquired through their medical school training as a whole, rather than the end of Emergency Medicine clerkship/rotation/course when answering the questions.

#### Phase 2: The selection of survey participants and the implementation of the survey (Modified Delphi Process)

The goal of the second phase was to select participants who are emergency physicians with expertise and experience in medical student education from a vast array of countries and to implement modified Delphi process via the survey. To do this, in August 2020, IFEM secretariat sent an online form asking all 52 IFEM voting member country representatives for up to five nominations. Three additional reminders were sent to non-responders on alternate weeks to increase the response rate.

In September 2020, one investigator sent an email, including a cover letter outlining the project and a link to the online survey, to invite nominees to the survey. Three additional reminders were sent to non-responders monthly to increase the response rate.

#### Phase 3: Analysis of collected data and the creation of IFEM recommendations (Nominal Group technique and Modified Delphi Method)

The goal of the third phase was to analyse the survey results, utilise the data to refine and update the ILO recommendations and to finalise the report. Phase 3 included seven steps. *First*, two investigators reviewed the survey results and sent them to the nominal group. The nominal group conveyed comments and feedback through email and a meeting. Three investigators drafted the initial report based on nominal group’s input. *Second*, the nominal group reviewed the draft report and provided written and verbal feedback which was then revised by three investigators. *Third*, one investigator sent the revised report to participants to ensure participants’ agreement and collect their feedback. *Fourth*, after required revisions were made, the nominal group examined the draft report and provided written feedback. *Fifth*, after further required revisions were made, the draft report was sent to CCEC for review and revised based on the written feedback. *Sixth,* the nominal group reviewed the report for a final time and provided written and verbal feedback through email and a meeting. *Seventh*, the CCEC and IFEM Board reviewed and approved the report finalised by the three investigators.

ILO recommendations were categorised into three tiers. For tier 1 ILOs, items with over 75% “must have” votes were considered. For Tier 2 ILOs, items that received a total of over 90% “must have” and “good to have” votes but below 75% “must have” votes were considered. The remaining items were considered as a guidance for Tier 3 ILOs as their value in relation to the medical school curriculum was dependent on health care system and setting. The final categories were decided upon based on the nominal group consensus. Items were synthesised and rephrased into understandable and applicable ILOs.

Tier 1 ILOs are recommended for all medical schools. Tier 2 ILOs are recommended for medical schools based on perceived local healthcare system needs and/or adequate resources. If a medical school has sufficient resources, they are encouraged to implement the Tier 2 ILOs. In resource-scarce environments, prioritisation should be based on perceived local healthcare system needs. Tier 3 ILOs should be considered for medical schools based on perceived local healthcare system needs and/or adequate resources. The rationale behind the latter tiers is that local healthcare system needs determine medical graduates’ immediate future roles in those settings. These recommendations are not prescriptive, and medical schools may integrate additional ILOs into their curricula depending on contextual needs.

## Data collection and analysis

The survey was created and distributed by using Google Forms, which is considered secure and practical [23]. The collected data was accessible by two investigators. After completion, all survey answers were extracted anonymously to a spreadsheet for analysis. Descriptive statistics were generated regarding the general characteristics of responding emergency medicine experts and their contexts, ILOs at the end of the medical school regarding emergency medicine education, emergency medicine specific principles and content areas unique to emergency medicine. The results are reported as numbers and percentages of responders.

## Results

### Participant & context information

Out of 112 invitees (CCEC members and IFEM member country nominees), 57 (50.9%) participants from 27 countries participated in the survey. The number and percentage of participants’ countries by IFEM regions are as follows: Asia (*n*=21, 36.8%), Europe (*n*=12, 21.1%), North America (*n*=12, 21.1%), Australasia (*n*=5, 8.8%), Africa (*n*=4, 7.0%), and Central and South America (*n*=3, 5.3%). The country response rates as a portion of IFEM member countries in each region were as follows: North America (*n*=4, 80%), Europe (*n*=6, 60%), Asia (*n*=12, 52.2%), Australasia (*n*=1, 50%), Africa (*n*=2, 40%), and Central and South America (*n*=2, 28.6%).

Out of 25 (48.1%) High Income Countries (HICs) and 27 (51.9%) Low- and Middle-Income Countries (LMICs) invited to the survey, 16 (64.0%) and 11 (40.7%) participated in the survey, respectively. Eighteen (31.6%) participants were from LMICs, while 39 (68.4%) participants were from HICs.

Forty-three (75.4%) participants have been practising emergency medicine for more than 5 years after speciality training, and 22 (38.6%) had more than 15 years of experience. Forty-nine participants (89.4%) were currently practising in an academic centre (*n*=43, 75.4%) or teaching hospital setting (*n*=8, 14.0%). Forty-two (73.7%) participants were serving both adult and paediatric patients. Forty-four (77.2%) participants have been involved with medical students’ emergency medicine training for more than five years in their career and 56 (98.2%) have been involved in medical students’ training in the last five years. Thirty-five (61.4%) participants have completed a form of training in medical education, including 2 (3.5%) PhD, 11 (19.3%) master’s degree, 8 (14.0%) diploma degree, and 14 (24.6%) certifications.

Fifty (87.7%) participants from 24 (88.8%) countries reported that they have a mandatory emergency medicine clerkship/rotation/course in their context, while 41 (71.9%) participants from 20 (74.1%) countries reported that they offer an elective clerkship/rotation/course. Two (3.5%) participants from 2 (7.4%) countries reported that they do not offer a mandatory or elective emergency medicine clerkship/rotation/course in their context. The clerkship/rotation/course durations ranged from one to two weeks to eight weeks or more. Fourteen (24.6%) participants from 11 (40.7%) countries reported that emergency medicine clerkship/rotation/course in their context was shorter than 2 weeks.

### Principles unique to emergency medicine

All participants found three out of six items relevant to some extent (must have and good to have votes equals to 100%) to medical students’ emergency medicine training, as shown in Table 1. The items that have 75% or more “must have” vote were:

1. emergency medicine prioritises care based on acuity and urgency (96.5%)
2. information, time and resource constraints may inhibit reaching a final diagnosis in the emergency department; therefore, emergency medicine prioritises differential diagnoses in a way to exclude life, organ and limb-threatening situations (87.7%)
3. in order to reach a timely and chief complaint-oriented diagnosis, emergency medicine uses a focused history and physical exam in undifferentiated patients (84.2%)
4. Emergency medicine provides 24-hour high-quality patient-centred healthcare to all patients with complex and undifferentiated complaints from any age group (75.4%).

**Table 1:** Principles unique to emergency medicine

**Item**	**Must have** **n (%)**	**Good to Have** **n (%)**	**Not Applicable** **n (%)**
Emergency Medicine prioritises care based on acuity and urgency.	55(96.5)	2(3.5)	-
Emergency medicine provides 24-hour high-quality patient-centred healthcare to all patients with complex and undifferentiated complaints from any age group.	43(75.4)	13(22.8)	1(1.8)
The acuity of the patients and continuous patient flow lead emergency physicians to time-constrained decision making in a resource-limited environment and develop a management plan for multiple patients simultaneously.	36(63.2)	19(33.3)	2(3.5)
In order to reach a timely and chief complaint-oriented diagnosis, emergency medicine uses a focused history and physical exam in undifferentiated patients.	48(84.2)	9(15.8)	-
Information, time and resource constraints may inhibit reaching a final diagnosis in the emergency department; therefore, emergency medicine prioritises differential diagnoses in a way to exclude life, organ and limb-threatening situations.	50(87.7)	7(12.3)	-
The emergency department provides a gateway for healthcare to the community, especially for the disadvantaged groups.	28(49.1)	27(47.4)	2(3.5)

#### Intended learning outcomes

All participants found five out of twenty items relevant to some extent (must have and good to have votes equals to 100%) to medical students’ emergency medicine training, as shown in Table 2. The items that have 75% or more “must have” vote were:

1. perform a focused assessment (history-taking, physical examination, investigation plan) on undifferentiated patients in the acute care setting (89.5%)
2. recognise in- and out-of-hospital cardiorespiratory arrest and perform basic and advanced life support (87.7%)
3. apply the fundamental principles related to emergency medicine (80.7%)
4. establish empathetic and effective professional relationships with patients and relatives, healthcare staff, and other stakeholders (80.7%).
5. re-evaluate the patient frequently for potential deterioration (78.9%).

**Table 2:** Intended learning outcomes

**Item**	**Must have** **n (%)**	**Good to Have** **n (%)**	**Not Applicable** **n (%)**
Apply the fundamental principles related to emergency medicine.	46(80.7)	11(19.3)	-
Recognise in- and out-of-hospital cardiorespiratory arrest and perform basic and advanced life support.	50(87.7)	7(12.3)	-
Prioritise the care of patients presenting to the ED	39(68.4)	18(31.6)	-
Perform a focused assessment (history-taking, physical examination, investigation plan) on undifferentiated patients in the acute care setting.	51(89.5)	6(10.5)	-
Demonstrate the principles of appropriate pharmaceutical and procedural therapeutic interventions in critical and emergent patients and seek timely expert support.	33(57.9)	22(38.6)	2(3.5)
Demonstrate the principles of appropriate initial therapy in lower acuity patients and provide a referral to the appropriate specialty.	27(47.4)	28(49.1)	2(3.5)
Re-evaluate the patient frequently for potential deterioration	45(78.9)	11(19.3)	1(1.8)
Describe the pre-hospital care’s value, importance and limitations in the healthcare system.	21(36.8)	30(52.6)	6(10.5)
Describe the importance of the continuum of therapy starting from pre-hospital care, through the emergency department and ending with an appropriate disposition of the patient and emergency medicine’s key position in it.	28(49.1)	27(47.4)	2(3.5)
Describe the principles of safe in- and out-of-hospital patient transfer.	25(43.9)	26(45.6)	6(10.5)
Describe the importance of the various emergency department members’ roles, function effectively as a team member, and coordinate multi-professional teams to ensure safe and efficient patient care.	27(47.4)	26(45.6)	4(7.0)
Recognise one’s limitations in the provision of emergency care.	41(71.9)	15(26.3)	1(1.8)
Critically appraise scientific literature using principles of evidence-based medicine	20(35.1)	29(50.9)	8(14.0)
Establish empathetic and effective professional relationships with patients and relatives, healthcare staff and other stakeholders.	46(80.7)	11(19.3)	-
Apply multi-tasking and time management skills to meet clinical and other professional standards.	19(33.3)	36(63.2)	2(3.5)
Inform and educate patients and relatives to optimise patient outcomes.	24(42.1)	32(56.1)	1(1.8)
Demonstrate the principles of basic audit projects and apply data to maintain and improve safe and effective practice and workplace environment.	9(5.8)	30(52.6)	18(31.6)
Document patient care by effective use of hospital information systems.	31(54.4)	23(40.4)	3(5.3)
Demonstrate the principles of safe and efficient prescribing.	35(61.4)	19(33.3)	3(5.3)
Apply ethical, professional, and legal principles related to emergency care context.	36(63.2)	19(33.3)	2(3.5)

#### Content

All participants found five out of thirteen items relevant to some extent (must have and good to have votes equals to 100%) to medical students’ emergency medicine training, as shown in Table 3. The items that have 75% or more “must have” vote were:

1. acute and/or critical illnesses and injuries (91.2%).
2. chief-complaint-based approach (86.0%).
3. approach to complex and undifferentiated patients (77.2%).
4. medical decision making in face of uncertainty, time- and resource-limitations (75.4%).

**Table 3:** Content unique to emergency medicine

**Item**	**Must have** **n (%)**	**Good to Have** **n (%)**	**Not Applicable** **n (%)**
Acute and/or critical illnesses and injuries	52(91.2)	5(8.8)	-
Approach to complex and undifferentiated patients	44(77.2)	13(22.8)	-
Caring for disadvantaged patients	20(35.1)	36(63.2)	1(1.8)
Chief-complaint-based approach	49(86.0)	8(14.0)	-
Death notification for sudden unexpected death	23(40.4)	30(52.6)	4(7.0)
Disaster management	9(15.8)	40(70.2)	8(14.0)
Environmental illnesses and injuries	22(38.6)	32(56.1)	3(5.3)
Injury prevention	16(28.1)	34(59.6)	7(12.3)
Intoxications	40(70.2)	17(29.8)	-
Medical decision making in face of uncertainty, time and resource limitations.	43(75.4)	14(24.6)	-
Pre-hospital care	16(28.1)	34(59.6)	7(12.3)
Resource utilisation	18(31.6)	34(59.6)	5(8.8)
Resuscitative team dynamics in undifferentiated critically ill or injured patients	40(70.2)	16(28.1)	1(1.8)

## Discussion

In this report, we aimed to identify emergency medicine related learning outcomes for medical student education based on available resources using consensus methodology. We received broad and diverse participation from colleagues with a good amount of educational expertise from all six IFEM regions with a mix of LMICs and HICs. The majority of participants reported that the emergency medicine rotation was mandatory; however, the duration of the clerkship/rotation/course varied. Participants expressed that medical students should learn the importance of prioritisation of clinical situations, management of undifferentiated patients and elimination of critical diagnoses within information, time, and resource-limited settings. Participants stated that medical students should be able to perform a focused assessment on undifferentiated patients, recognise cardiac arrest and perform BLS and ACLS and apply fundamental principles of emergency medicine. They also indicated that contributions of emergency physicians/medicine as a speciality were most important for teaching the topics of acute and critical illnesses/injuries and the chief-complaint-based approach.

There is a wide variation in how emergency medicine clerkships/rotations/courses are implemented across the world [7, 10, 24, 25]. Our survey shows the presence of settings that implement mandatory-only, elective-only and both options as emergency medicine clerkships/rotations/courses. Additionally, clerkship/rotation/course durations range extensively from one to two weeks to eight weeks or more. Moreover, even though our survey does not represent the totality of countries, 40.7% of responding countries seem to have at least one institution that offers less than a three-week clerkship/rotation/course. The available literature supports a mandatory clerkship/rotation/course of a minimum of four weeks in senior years to prepare students with adequate educational opportunities [4, 25–31]. Institutions may also consider additional mandatory or elective clerkships/rotations/courses in earlier years to support vertical integration and students aiming for more in-depth learning or a career in emergency medicine [24, 32]. Ultimately, the goal of all integration should be to ensure students gain the competencies required by the immediate future work [33].

Emergency medicine operates on slightly different principles compared with other medical disciplines. Most participants acknowledged that all principles seen in Table 2 are important to some extent. More precisely, the majority of participants considered teaching emergency medicine’s acute-care, chief-complaint-based approach that prioritises the worst-case scenarios essential. Similarly, the literature supports that a chief-complaint-based approach helps students to develop diagnostic and therapeutic decision-making skills [25, 34]. Educators should teach students how to approach chief complaints in the sense that they will prioritise excluding worst-case scenarios and considering the most common emergencies with appropriate management [4, 5]. Interestingly, despite emergency departments’ role as a safety net in some settings [35], only half of the participants considered teaching emergency medicine as a gateway to healthcare for disadvantaged groups essential to the curriculum for medical students. This finding may be caused by different contextual expectations relating to the role of emergency departments based on systemic necessities rather than emergency medicine’s primary objectives [36]. Overall, these principles help students understand how emergency medicine functions in the healthcare system.

Medical schools should ensure that medical graduates have acquired a certain set of knowledge, skills, and attitude regarding basic emergency care. In our survey, potential ILO items with the highest consensus were associated with basic clinical skills and communication. These learning outcomes were aligned with internationally recognised medical school curriculum recommendations [21, 37, 38]. However, additional roles emphasised in these frameworks, such as the ability to understand evidence-based medicine and quality assurance, were less prioritised as ILOs in the results of our survey. This may mean that emergency medicine educators consider acquisition of these roles less specific to emergency medicine education. Additionally, fewer participants considered the treatment and prehospital care-related items essential to emergency medicine curriculum in medical school. This may be a consequence of the fact that medical graduates without further postgraduate training undertake different roles in diverse systems ranging from non-clinical jobs to independent clinical practice [7, 14, 15, 39, 40].

Emergency medicine distinguishes itself from other disciplines through some unique content areas. Regarding the medical school curriculum, participants considered acute management of undifferentiated patients an essential content area to teach medical students. Notably, most deemed sensitive and organisational items, such as death notification and resource utilisation, a potentially valuable but less essential part of the medical school curriculum. Similarly, content areas specific to emergency medicine subspecialties, such as disaster medicine and pre-hospital care were less prioritized in the medical school curriculum. This can be explained by the fact that such areas are covered by a range of professionals from non-physician healthcare professionals to sub-specialised emergency physicians in different settings [41].

Medical education should focus on reaching general learning outcomes rather than separate goals of each discipline [42]. In this sense, emergency medicine related outcomes should be aligned with the general outcomes of the medical school’s curriculum, and teaching and learning activities should be integrated horizontally and vertically in a way to reach these goals. In this document, we aimed to identify emergency medicine related acquisitions at the end of the medical school rather than a specific year, course or clerkship. These acquisitions should be broken down among years and specific courses throughout the medical school curriculum. A stepwise approach starting in the early years by teaching fundamental knowledge, skills and attitude and building up each year and course with increasing exposure to more authentic clinical environments helps with a more efficient education [32, 43, 44]. Such an educational strategy requires an aligned collaboration among diverse medical disciplines including pre-clinical and clinical years [45]. In this larger plan, emergency medicine clerkships/rotations/courses should be one of the steps where students are exposed to real patient encounters and educational opportunities in clinical environments [46–50].

### Recommendations

In light of the international opinions from the survey and literature review, IFEM CCEC undergraduate emergency medicine curriculum update task force updated the ILO recommendations as shown in Table 4.

**Table 4:** IFEM ILO recommendations for medical student education

**Tier 1 ILOs: Recommended for all medical schools**	
At the end of medical school, medical graduates should be able to:	
1	Prioritise patients from any age group based on the acuity and urgency of clinical situation
2	Perform a focused assessment (history-taking, physical examination, investigation plan) on undifferentiated patients in the acute care setting
3	Apply chief-complaint-based approach to prioritize the worst-case scenarios or common life, organ, and limb threatening diagnoses
4	Recognise in- and out-of-hospital cardiorespiratory arrest and perform basic and advanced life support
5	Apply the principles of common pharmaceutical and procedural therapeutic interventions in critical and emergent patients and seek timely expert support
6	Demonstrate empathetic and effective professional relationships and communication with patients and relatives, healthcare staff, and other stakeholders
7	Apply ethical, professional and legal principles related to emergency care context
**Tier 2 ILOs: Recommended for medical schools based on perceived local healthcare system needs and/or adequate resources.**	
At the end of medical school, medical graduates should be able to:	
1	Function effectively as a team member in a multi-professional team
2	Apply the principles of appropriate initial therapy in lower acuity patients and provide an appropriate referral to other specialties
3	Apply a management plan for multiple patients simultaneously
4	Document patient care by effective use of hospital information systems
5	Apply the principles of safe and efficient prescribing
6	Describe the principles of safe in- and out-of-hospital patient transfer
7	Counsel and educate patients and relatives effectively
**Tier 3 ILOs: Should be considered for medical schools based on perceived local healthcare system needs and/or adequate resources**	
At the end of medical school, medical graduates should be able to:	
1	Recognise vulnerable populations and adjust care according to their specialised needs
2	Apply basic emergency care in a pre-hospital setting with adherence to the continuum of care
3	Perform basic emergency management in disaster response teams
4	Critically appraise scientific literature using principles of evidence-based medicine
5	Perform basic audit projects and apply data to maintain and improve safe and effective practice and workplace environment
6	Adapt medical care according to available resources
7	Demonstrate knowledge to inform less knowledgeable others on how to prevent frequent injuries

Tiered ILO recommendations represent the consensus of international emergency medicine education experts and aim to establish fundamental global standards. Tier 1 ILOs are considered central to emergency medicine and are recommended for all medical schools. It should be noted that an ILO being in Tier 2 or Tier 3 implies that it applies varyingly to settings with different resource levels or is perceived as less associated with acute care-related outcomes, rather than its importance in emergency medicine. Therefore, if the resources allow or contextual pressures require, institutions should aspire to integrate all three tiers into their medical school curriculum. Moreover, institutions are free to add additional ILOs as necessary. We believe that updated recommendations facilitate addressing different contextual needs with a resource-neutral approach and increase feasibility in diverse healthcare systems and settings.

## Strengths and limitations

This report has several strengths. We gathered opinions of a diverse group of emergency physicians with a structured consensus method. The fact that the majority of participants had theoretical and practical experience in education increased the validity of the data collected. We believe that the range of countries represented in our survey and nominal group increases the generalizability of our results and recommendations. Finally, a nominal group of international emergency medicine leaders interpreted the data and provided setting-neutral learning outcomes, increasing the recommendations’ applicability.

This report has several limitations. The response rate of the survey was around 50%. Whilst a higher response rate would be favourable, this response rate can be considered acceptable in such an international process. We invited all IFEM member countries to the survey. Even though the numbers of responding HICs and LMICs were similar, respondents from HICs outweighed those from LMICs. This has two major reasons: First, although the survey was sent equally to all countries, more participants per country responded to the survey in HICs compared to LMICs. Second, the representation of HICs and LIMCs in the CCEC was not equal at the time of the piloting process. Although we collected participants’ opinions from diverse settings, our sampling method does not allow us to offer regionalised recommendations. As a result of each region and setting having unique needs and resources, more focused studies are recommended to address these. Finally, the evolving nature of emergency medicine and medical education will require future updates to this document, such as integrating advanced technologies into the medical school curriculum.

## Conclusion

In this document, we reviewed the first IFEM model curriculum for medical student education in emergency medicine in light of the contemporary literature and international expert perspectives to provide setting- and resource-neutral ILO recommendations. We received input from all IFEM regions, which was interpreted by an international group of emergency medicine education experts by consensus methods. We provided recommendations that can be used in various settings to set the global minimum standards of the outcomes regarding emergency medicine education in medical schools.

The understanding of emergency medicine and medical education constantly evolves, requiring regular updates on existing curricula. As a part of this update, this document provides emergency medicine learning outcomes for medical students, shaped by current medical education and emergency medicine trends. These updated IFEM recommendations can be a model framework for many countries and institutions to establish, promote and improve a standardised emergency medicine education in medical schools. However, the local context for emergency medicine education depends on the national and regional healthcare policies, public and governments’ conception of emergency medicine and departments and educational and clinical resources. Therefore, countries and institutions should take contextualised needs into account when integrating IFEM ILO recommendations into their curriculum.

## Data Availability

All data generated or analysed during this study are included in this published article.

## Abbreviations

ACLS: Advanced Cardiac Life Support
BLS: Basic Life Support
CCEC: Core Curriculum and Education Committee
ED: Emergency Department
HICs: High Income Countries
IFEM: The International Federation for Emergency Medicine
ILO: Intended Learning Outcome
LMICs: Low- and Middle-Income Countries
